# Benign Adrenocortical Tumors and the Detection of Nonadrenal Neoplasia

**DOI:** 10.1155/2019/9035407

**Published:** 2019-11-03

**Authors:** Meng Hao, Miguel Angel Luque-Fernandez, Diana Lopez, Kathryn Cote, Jessica Newfield, Molly Connors, Anand Vaidya

**Affiliations:** ^1^Department of Endocrinology, Diabetes and Hypertension, Brigham and Women's Hospital, Boston, Massachusetts 02115, USA; ^2^Harvard Medical School, Boston, Massachusetts 02115, USA; ^3^Harvard School of Public Health, Boston, Massachusetts 02115, USA; ^4^London School of Hygiene and Tropical Medicine, London, UK; ^5^Biomedical Research Institute of Granada, Non-communicable Disease and Cancer Epidemiology Group, Andalusian School of Public Health, University of Granada, Granada, Spain

## Abstract

**Context:**

Patients with adrenocortical tumors have been frequently observed to have nonadrenal neoplasia.

**Objective:**

To investigate whether patients with benign adrenocortical tumors have a higher likelihood of having nonadrenal neoplasia detected.

**Design and Participants:**

Case-control study of patients with benign adrenocortical tumors (cases; *n* = 400) and normal adrenal glands (controls; *n* = 400), who underwent repeated abdominal cross-sectional imaging.

**Main Outcomes:**

Primary analyses: association between case-control status and benign abdominal neoplasia detected via cross-sectional imaging. Secondary analyses: association between case-control status and tumors detected via other imaging modalities.

**Results:**

The mean interval of abdominal imaging was 4.7 (SD = 3.8) years for cases and 5.9 (4.8) years for controls. Cases were more likely to have detected intraductal papillary mucinous neoplasms (IPMNs) of the pancreas (8.5% vs. 4.5%, adjusted OR = 2.22, 95% CI (1.11, 4.63)) compared with controls. In secondary analyses, cases were more likely to have detected thyroid nodules (25.5% vs. 17.0%, adjusted OR = 1.77, 95% CI (1.15, 2.74)), hyperparathyroidism or parathyroid adenomas (3.5% vs. 1.3%, adjusted OR = 3.00, 95% CI (1.00, 11.64)), benign breast masses (6.0% vs. 3.3%, adjusted OR = 3.25, 95% CI (1.28, 8.78)), and benign prostatic hyperplasia (20.5% vs. 5.3%, adjusted OR = 3.20, 95% CI (1.14, 10.60)). Using a composite outcome, cases had higher odds of detection of the composite of IPMN, thyroid nodules, parathyroid tumors, benign breast masses, and prostate hyperplasia (adjusted OR = 2.36, 95% CI: 1.60, 3.50) when compared with controls.

**Conclusions:**

Patients with benign adrenocortical tumors had higher odds of detected pancreatic IPMN, as well as thyroid nodules, parathyroid tumors, benign breast masses, and prostate hyperplasia compared with patients with normal adrenal glands. These associations may have important implications for patient care and healthcare economics, regardless of whether they reflect incidental discoveries due to imaging detection or frequency bias, or a common risk for developing multiple neoplasia.

## 1. Introduction

Benign adrenocortical tumors are common incidental findings on abdominal computed tomography (CT) and magnetic resonance imaging (MRI) scans [1]. As imaging technologies are increasingly used in clinical evaluations, these incidental findings are becoming more prevalent. While most adrenocortical tumors are benign and presumed to be the consequence of somatic alterations, there are rare instances when they are driven by heritable genetic alterations. For instance, adrenocortical tumors are well-known manifestations of rare and classical genetic tumor syndromes such as Li-Fraumeni, Beckwith–Wiedemann, multiple endocrine neoplasia type 1, familial adenomatous polyposis, and Carney complex and are seen with newer inheritable mutations. These classical tumor syndromes are associated with varying morphologies of adrenocortical neoplasia including hyperplasia, adenomas, and carcinomas, thus linking the development of adrenal neoplasia with a variety of extra-adrenal neoplasms via inherited mechanisms.

While the exact cause and risk factors for the high incidence of adrenocortical tumors are not fully resolved, genetic susceptibilities or common environmental exposures may increase the overall risk of neoplasia in multiple organs. For example, previous research on intraductal papillary mucinous neoplasms (IPMNs) has shown that patients with IPMNs have a higher risk for concomitant extrapancreatic neoplasms in the absence of any syndromic diagnosis [2–4]. Similarly, patients with adrenal incidentalomas have been observed to have a higher prevalence of thyroid nodules [5], and patients with pituitary adenomas may be at a higher risk for developing primary tumors at other sites [6–11]. Data from the nationwide Swedish Family-Cancer Database which examined the risk of subsequent primary neoplasms after the occurrence of an endocrine gland tumor [12] found that the risk for a second endocrine tumor was substantially higher following the first [12]; thereby, suggesting that the development of a benign endocrine tumor may increase the likelihood of developing subsequent tumors [12].

Herein, we sought to systematically investigate whether patients with an adrenocortical tumor, but without any known syndromic or inheritable diagnosis, were more likely to have detected other benign abdominal or extra-abdominal neoplasia. We conducted a case-control study among patients who underwent repeated abdominal cross-sectional imaging over time to assess whether having a benign adrenocortical tumor was associated with a higher risk of detection of other benign abdominal, and extra-abdominal, neoplasia when compared with patients with normal adrenal glands.

## 2. Methods

### 2.1. Study Population

**Figure 1:**
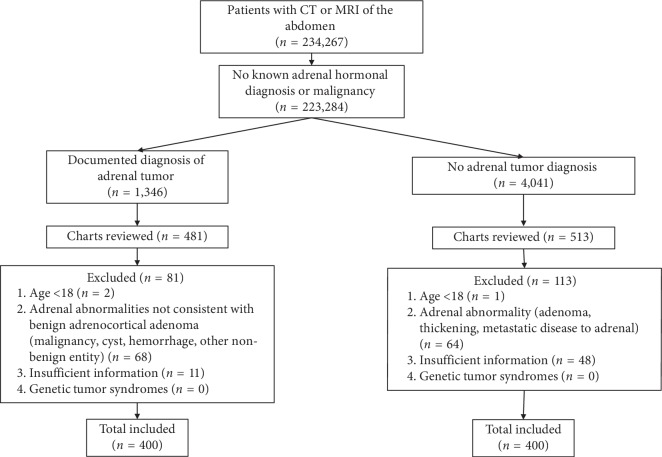
Study participant inclusion process. Participants were selected from patients who underwent abdominal CT or MRI at our institution.

We designed a traditional case-control study with a cumulative sampling design. Cases and controls were both drawn from a single institutional research registry of all patients from Brigham and Women's Hospital, Massachusetts General Hospital, and their affiliated partner hospitals (Figure 1). We searched this registry for any patient with abdominal CT or MRI assessments from 1989 to 2016 (*n* = 234, 267) and excluded patients with known or possible adrenal malignancy or adrenal hormonal diagnosis such as primary aldosteronism, Cushing's syndrome, congenital adrenal hyperplasia, and pheochromocytoma. Cases were selected from those with diagnosis codes for benign adrenocortical tumor (*n* = 1,346), while three times as many controls were selected from those with no adrenal tumor (*n* = 4,041). We completely reviewed medical records from an arbitrary sample of 481 consecutive cases and 513 controls, with no efforts to match or pair selections, and excluded patients with insufficient information (lack of documentation of comprehensive clinical evaluations) or if they were <18 years old. Comprehensive clinical evaluations included any annual physical examination, primary care or general internal medicine visits, medical subspecialty consultations, and preoperative anesthesia consultations. We also attempted to exclude anyone with a diagnosis of any known genetic syndrome associated with adrenocortical adenoma, including multiple endocrine neoplasia type 1, Li-Fraumeni, McCune Albright syndrome, Carney complex, Beckwith-Wiedemann syndrome, familial adenomatous polyposis, and congenital adrenal hyperplasia; however, after careful review of medical records, no participant had documentation of such diagnoses. We reviewed each patient's radiology reports and considered cases as having a benign adrenocortical tumor if the tumor had a lipid-rich attenuation (<10 Hounsfield Units) on unenhanced CT imaging, high contrast washout on CT imaging (if available), a marked drop in signal intensity on MRI imaging suggestive of a lipid-rich adenoma, and/or other descriptors that are suggestive of a benign tumor, such as “benign” or “adenoma” or “myelolipoma.” We excluded any potential cases with an adrenal abnormality not consistent with a benign adrenocortical tumor (potential malignancy, cyst, hemorrhage, and other nonbenign entity) and any potential controls with evidence of an adrenal abnormality (adenoma, thickening, metastatic disease, cyst, and hemorrhage) for a final study population of 400 cases with benign adrenocortical tumors and 400 controls with no evidence of any adrenal abnormality.

### 2.2. Assessment of Baseline Characteristics

We collected demographic information (age, sex, race, weight, height, and derived body mass index (BMI) in kg/m^2^), smoking status, and hospital affiliation of primary care providers from each patient's electronic medical record. We then determined the first abdominal imaging study that demonstrated benign adrenocortical tumor or normal adrenal glands for cases and controls, respectively. This was considered the baseline timepoint for adrenal morphology assessment. We collected data on cardiovascular comorbidities (hypertension, hyperlipidemia, coronary artery disease, myocardial infarction, and prediabetes or type 2 diabetes mellitus) and medications from baseline. Hyperlipidemia was defined as a documented diagnosis and/or a low-density lipoprotein cholesterol level of ≥150 mg/dL (3.89 mmol/L). Composite diabetes was defined as having either a documented diagnosis of prediabetes or type 2 diabetes mellitus. Any patient with ≥2 documented hemoglobin A1c value of 5.7% to 6.4% (no hypoglycemic agent other than metformin) was considered to have prediabetes, and any patient with ≥2 documented hemoglobin A1c value ≥ 6.5% was considered to have type 2 diabetes mellitus.

We identified the most recent abdominal CT/MRI for all patients and calculated the interval of imaging follow-up defined by the time elapsed from the initial to the most recent abdominal imaging study. The interval of imaging follow-up was used as a proxy of the opportunity to detect the presence of extra-adrenal abdominal neoplasia over time.

### 2.3. Primary Outcome Assessment: Abdominal Neoplasia Detected via Abdominal CT/MRI

We individually reviewed each patient's entire collection of abdominal CT or MRI reports in the interval of imaging follow-up for benign tumors of the major abdominal organs assessed including the hepatobiliary system, pancreas, spleen, and kidneys (Table 1). Since all included participants had repeated abdominal cross-sectional imaging studies over time, the opportunity to detect these abdominal tumors among cases and controls was similar and the risk for imaging detection bias deemed to be minimal.

### 2.4. Secondary Outcomes Assessments: Extra-Abdominal Benign and Malignant Tumors

We recorded the detection of other benign neoplasia of extra-abdominal organs from all other available imaging modalities and the colon from colonoscopy reports (Table 2). We also reviewed each patient's medical records for any clinical diagnoses of malignant tumors or cancer based on physician/provider documentation, reference to pathology reports, or ICD coding. The use of other imaging modalities besides abdominal CT/MRI was not standardized, and the number of additional imaging studies could not be reliably quantified using our open electronic medical health records system; therefore, our study design could not confidently differentiate whether extra-abdominal tumor detection was the cause or consequence of imaging selection bias (for example, patients with adrenocortical tumors may undergo more extra-abdominal imaging studies than those without adrenocortical tumors and thereby a greater discovery rate of incidental findings) or true differences in tumor incidence.

### 2.5. Statistical Analysis

We present patients' baseline and demographic characteristics by case-control status using frequencies and percentages for categorical variables and means and standard deviations for continuous variables. Univariate differences between baseline and demographic characteristic by case-control status are presented using chi-square, Fisher's exact, and Student's *t*-test tests. The associations between case-control status and individual nonadrenal neoplasia were evaluated by computing univariate odds ratios and their respective 95% confidence intervals (CI) and by fitting multivariate logistic regression models adjusted for age, sex, race, BMI, smoking status, imaging interval duration (defined as the time interval between first and last available abdominal imaging study), and cardiovascular comorbidities (hypertension, hyperlipidemia, composite diabetes, coronary artery disease, and myocardial infarction). When assessing the risk for thyroid nodules, we also conducted sensitivity analysis restricted to only thyroid nodules that were ≥1 cm and thyroid nodules that had cytology confirmation of benign cells. Statistical analyses were performed by using R version 3.0.2 (R Foundation for Statistical Computing, Vienna, Austria).

## 3. Results

### 3.1. Baseline Demographics and Characteristics

Demographic and clinical data of cases and controls are presented in Table 3. Cases were more likely to be male, white, and older in age and have a higher BMI and prevalence of diabetes compared with controls. Both cases and controls had approximately 5 years of abdominal cross-sectional imaging follow-up to examine for abdominal tumors, although cases had a slightly shorter opportunity to detect tumors (Table 3).

### 3.2. Primary Outcome: Benign Abdominal Neoplasia Detected via Abdominal CT/MRI

The adjusted odds ratios for each benign nonadrenal tumor type detected via abdominal cross-sectional imaging are presented in Table 4. Cases had significantly higher odds of having a pancreatic IPMN (adjusted OR = 2.22, 95%CI: 1.11, 4.63). There were nonsignificant trends, further limited by small sample sizes, suggesting that cases may be more likely to have hepatic cysts, splenic hemangioma or cysts, renal angiomyolipoma, and renal cysts when compared with controls.

### 3.3. Secondary Outcomes Assessments: Extra-Abdominal Benign and Malignant Tumors

The adjusted odds ratios for each extra-abdominal tumor type detected via other modalities are presented in Table 5. Cases had significantly higher odds of having a thyroid nodule (adjusted OR = 1.77, 95%CI: 1.15, 2.74), parathyroid tumors and hyperparathyroidism (adjusted OR = 3.00, 95%CI: 1.00, 11.64), benign breast tumors (adjusted OR = 3.25, 95%CI: 1.28, 8.78), and benign prostatic hyperplasia (adjusted OR = 3.20, 95%CI: 1.14, 10.60). In sensitivity analyses restricted to only thyroid nodules that were ≥1 cm and thyroid nodules that had cytology confirmation of benign cells, cases still had higher odds of having thyroid nodules compared with controls, although the sample size was much smaller and confidence intervals crossed 1.00 (adjusted OR = 1.72, 95%CI: 0.73, 4.13 and adjusted OR = 2.12, 95%CI: 0.87, 5.50, respectively). There were no differences in the risk for any malignant tumors or cancer (Table 6).

### 3.4. Composite Outcome

When using a composite outcome for extra-adrenal neoplasia, having a benign adrenocortical tumor (case status) was associated with significantly higher odds of detection of the composite of IPMN, thyroid nodules, parathyroid tumors, benign breast masses, and prostate hyperplasia (adjusted OR = 2.36, 95%CI: 1.60, 3.50) when compared to patients with normal adrenal glands (control status).

## 4. Discussion

It is common for adrenocortical tumors to be detected serendipitously with abdominal imaging performed for other reasons. While most of these tumors are benign and sporadic, adrenocortical tumors are also phenotypic manifestations of certain classical inheritable tumor syndromes. The observations of our current study suggest that even in the absence of known syndromic neoplasia, patients with benign-appearing adrenocortical tumors may be more likely to have detected IPMNs, as well as benign neoplasia of the thyroid, parathyroid, breast, and prostate. Whether these tumors of different organs were incidentally detected due to imaging performance bias or imaging detection bias, or are causally linked through a common pathway, could not be assessed with the current study design; however, the ultimate observation has important implications for the clinical care of patients, *regardless of the reason* for the detection difference. The possibility that patients with an incidentally detected adrenocortical tumor are more likely to have detected extra-adrenal tumors (either due to excessive scrutiny of available images or via additional imaging studies ordered) could have major implications for healthcare spending and patient care. Alternatively, the possibility that there is a casual link between adrenocortical tumors and these extra-adrenal tumors, as discussed below, may also have important implications, given how common adrenocortical tumors are.

The association between having adrenocortical tumors and IPMNs was robust and independent of numerous potential confounders. Both cases and controls underwent repeated cross-sectional abdominal imaging over time wherein controls were imaged over a longer interval of time, thereby providing greater opportunity to detect potential tumors. In this regard, the observation that IPMNs are more likely to be detected among cases (patients with adrenocortical tumors) is unlikely to be due to detection or imaging bias. In this regard, our findings support prior studies demonstrating that having one endocrine neoplasm may increase the risk of having other tumors [12] and that having an IPMN is associated with having other nonpancreatic tumors [2–4]. In contrast, our secondary observations that adrenocortical tumors were also associated with a higher likelihood of having benign tumors of the thyroid, parathyroid, breast, and prostate were less robust. The study was designed with standardized abdominal cross-sectional imaging between cases and controls, and therefore, the confidence and consistency in the detection of abdominal tumors was high. However, the study design could not standardize the indications or frequency/number of imaging studies focused on the neck, chest, pelvis, and other sites. Since we could not reliably account for these factors, it is possible that the association of having tumors of the thyroid, parathyroid, breast, and prostate among individuals with adrenocortical tumors may be due to imaging performance and/or detection bias, wherein patients with adrenocortical tumors could have undergone more imaging studies with closer scrutiny for other tumors, rather than a true causal link.

Regardless of the mechanistic explanation for the association, these observations highlight an important clinical phenomenon: patients with adrenocortical tumors are either more likely to have detected, or more likely to develop, benign neoplasia of the pancreas, thyroid, parathyroid, breast, and/or prostate. It may certainly be possible that the incidental detection of one tumor, directly or indirectly, leads to more imaging procedures that result in the detection of other incidental tumors (a factor our study design could not reliably quantify). Whether this is of benefit to patients (i.e., early detection of potentially harmful processes) or a burden to patients and healthcare spending (i.e., excessive detection of benign and clinically innocuous tumors) is an important query for the medical field to consider. Future work should therefore focus on elucidating whether the imaging detection of these tumors are a boon or burden on patient care and healthcare economics and/or whether there is a common genetic or other causal link for these observations.

Possible biological explanations for our findings include potential genetic predispositions involved in a common neoplastic pathway may lead to greater risk of developing multiple benign neoplasia. These genetic variants may involve known genes that are already implicated in rare tumor syndromes (i.e., *MEN1* and *TP53*) or novel and complex polygenic variation that is currently not well understood. It has been well demonstrated that there is a high rate of *β*-catenin alterations in adrenocortical tumors suggesting that activation of the *Wnt*/*β*-catenin signaling pathway may play a major role in adrenocortical tumorigenesis [13–15]; however, this abnormality also exists in nonadrenal tumors. In a study by Chetty et al., 7 of 18 (39%) cases of benign and malignant IPMNs displayed abnormal *Wnt*-signaling pathway with abnormal localization of *β*-catenin [16]. Björklund et al. demonstrated that all 47 (100%) analyzed parathyroid tumors, and all 84 (100%) parathyroid tumors in a subsequent study, demonstrated abnormal *β*-catenin accumulation [17, 18]. Similarly, Whitaker et al. found abnormal nuclear accumulation of *β*-catenin in 40% of the 80 samples of benign prostatic hyperplasia [19]. Hence, germline or other acquired abnormalities in the *Wnt*/*β*-catenin pathway may represent one potential cause of acquiring a higher collective risk for developing these tumors.

Another explanation may be that there exists a higher frequency of genetic and phenotypic variants of the known classical tumor syndromes within the general population. Vouillarmet et al. reported a patient with a hormonally active adrenocortical tumor before any symptoms of familial adenomatous polyposis appeared [20]. Similarly, Talaei et al. reported a patient presenting with a right adrenocortical tumor and an incidentally discovered pituitary adenoma who was subsequently diagnosed with Carney complex suggesting potentially greater phenotypic heterogeneity [21]. While these are isolated case reports, it may be possible that there are individuals without the “classical,” or severe, presentations of the known inherited neoplasia syndromes, or with less penetrant manifestations that evade formal diagnosis.

Other possible explanations may include environmental risk factors leading to increased risk for multiple benign neoplasia. While we did not detect a significant difference in smoking history, we could not evaluate other lifestyle habits such as physical activity, diet, alcohol use, and other environmental variables. Our data show significantly greater BMI and diabetes prevalence status in individuals with adrenal tumors. Previous studies have examined the relationship between obesity, insulin resistance, and tumorigenesis. While incompletely understood, insulin resistance may promote tumorigenesis through increased levels of insulin, insulin-like growth factors, and sex steroids and their role in energy intake, increased cellular proliferation, and suppression of apoptosis [22–27]. Studies have also suggested that high BMI and adiposity result in greater inflammation that may promote tumorigenesis [25–29], and associations between high BMI and diabetes with increased risk for colon, liver, pancreatic, kidney, and endometrial cancer have been reported [25–33]. While these studies examined malignant tumors, it may be possible that the hormonal milieu and cellular environment of individuals with diabetes and high BMI may also increase the risk for benign neoplasia and hyperplasia.

Our study must be interpreted within the context of our study design. Bias is an important consideration in case-control studies. We attempted to limit selection bias by using arbitrary selection of consecutive patients from the same institutional database. Differences in the imaging frequency of abdominal and extra-abdominal organs represent another source of bias, as discussed extensively above. The frequency of abdominal imaging was not standardized; however, the use of cross-sectional abdominal imaging was required, and the imaging interval over time permitted ample opportunity to detect abdominal tumors in both cases and controls. In fact, we observed that even though patients with normal adrenal glands (controls) were imaged over a longer interval of time, patients with adrenal tumors (cases) had significantly higher odds of having abdominal pancreatic IPMNs. We also observed nonsignificant trends suggesting a higher risk for several other benign tumors and masses; larger sample sizes would be needed to ascertain whether these trends are suggestive of true associations. Given the observational and retrospective nature of this study, the indications for various types of diagnostic imaging in our secondary analyses were not controlled, and it is possible that cases were subjected to more imaging opportunities than controls. We could not confidently quantify the number of imaging tests each patient underwent since patients may have undergone some testing outside of our health system; however, even if cases underwent more frequent imaging, the interpretations of our findings still implicate that patients with adrenocortical tumors have a higher likelihood of detection of benign extra-adrenal neoplasia. Our study was not designed to determine the directionality or temporal relationship between development of adrenal neoplasia and extra-adrenal neoplasia, or the involvement of another factor (genetic or environmental). Therefore, we cannot make firm conclusions regarding the direction, timing, or pathogenic cause of these findings. Because our observations were based on standard clinical care, patients did not undergo repeated evaluations for subclinical hypercortisolism, or other adrenal hormone excess, which can contribute to cardiometabolic diseases. In addition, while patients in our study did not have clinical diagnoses to indicate known genetic tumor syndromes, we did not have the ability to conduct systematic genetic testing to confirm this, nor evaluate potential genetic contributions to our findings. Lastly, while we adjusted for all potential covariates available to us, there remains a risk for residual confounding.

In conclusion, we observed that having benign adrenocortical neoplasia was associated with a significantly higher likelihood of detection of pancreatic IPMN and other benign neoplasia or hyperplasia of the thyroid, parathyroid, breast, and prostate, in the absence of a diagnosis of a known genetic tumor syndrome. It is possible that these findings are the result of detection or imaging bias resulting in increased frequency of incidental tumor detection, mild or common pathogenic variants of rare genetic syndromes in the population, and/or unrecognized environmental exposures. *Regardless of the underlying cause of this observation*, these findings have important implications in determining the oncologic risk profile and clinical management of patients found to have adrenocortical adenomas.

## Figures and Tables

**Table 1:** Outcome assessments on abdominal cross-sectional imaging.

Organ/gland	Benign neoplasia	Detection modality
Hepatobiliary	Hepatobiliary hemangioma or adenoma Hepatic cysts	Abdomen CT/MRI
Pancreas	Intraductal pancreatic mucinous neoplasm	
Spleen	Splenic hemangioma and cysts	
Kidneys	Renal angiomyolipoma Renal cysts	

**Table 2:** Outcome assessments on other imaging modalities.

Organ/gland	Benign neoplasia	Detection modality
Meninges	Meningioma	Brain MRI
Pituitary	Pituitary adenoma	Head CT
Thyroid	Thyroid nodule	Thyroid ultrasound head, neck, or chest CT
Parathyroid	Hyperparathyroidism or Parathyroid adenoma	Parathyroid scan with SPECT CT Parathyroidectomy and pathology PTH labs and clinical diagnosis
	
Breasts	Fibroadenoma	
	Papilloma	Breast biopsy pathology
	Other benign neoplasm	Surgical excision pathology
	Breast cysts	
Colon	Colon adenoma	Colonoscopy
Prostate	Benign prostatic hyperplasia	Clinical diagnosis
		Pelvic CT/MRI
Adipose	Lipoma	Clinical diagnosis
		CT/MRI
Uterus	Fibroid	Pelvic CT/MRI Pelvic ultrasound
	Endometrial polyp	
Cervix	Cervical polyp	
Ovary	Ovarian cyst	

**Table 3:** Baseline demographic and clinical characteristics of cases and controls.

Characteristic	Cases (adrenocortical tumor)	Controls (normal adrenal)	*P* value
Patients, *n*	400	400	
Mean age, years (SD)	62.7 (12.9)	58.3 (14.5)	<0.001
Sex			
Female	268 (67.0%)	306 (76.5%)	0.001
Male	132 (33.0%)	94 (23.5%)	
Race			
White	294 (73.5%)	257 (64.3%)	0.043
Black	29 (7.25%)	37 (9.3%)	
Hispanic	16 (4.0%)	26 (6.5%)	
Others	61 (15.25%)	80 (20.0%)	
Institutional primary care provider	237 (59.3%)	251 (62.8%)	0.31
Mean BMI, kg/m^2^ (SD)	29.5 (6.9)	28.5 (7.0)	0.047
Interval of abdominal cross-sectional imaging^*∗*^, years (SD)	4.7 (3.8)	5.9 (4.8)	0.007
Smoking status			
Nonsmoker	173 (43.3%)	216 (54.0%)	0.37
Current or past smoker	227 (56.7%)	184 (46.0%)	
Comorbidity			
Hypertension	242 (60.5%)	193 (48.2%)	0.41
Diabetes or prediabetes	107 (26.7%)	71 (17.8%)	0.04
Hyperlipidemia	207 (51.7%)	169 (42.2%)	0.92
Coronary artery disease	60 (15.0%)	40 (10.0%)	0.62
Myocardial infarction	29 (7.2%)	16 (4.0%)	0.50

*Note*. ^*∗*^Interval of imaging defined as the time period between the initial and most recent cross-sectional abdominal imaging study.

**Table 4:** Risk for benign abdominal neoplasia detected via abdominal cross-sectional imaging.

Benign abdominal neoplasm	Cases (adrenocortical tumor)	Controls (normal adrenal)	OR (95% CI)	AOR (95% CI)^*∗*^
*n*	400	400		
Hepatobiliary hemangioma or adenoma	26 (6.5%)	19 (4.8%)	1.39 (0.76, 2.59)	1.30 (0.61, 2.79)
Hepatic cyst	109 (27.3%)	91 (22.8%)	1.27 (0.93, 1.75)	1.44 (0.98, 2.17)
Intraductal pancreatic mucinous neoplasm	34 (8.5%)	18 (4.5%)	1.97 (1.11, 3.62)	2.22 (1.11, 4.63)
Splenic hemangioma and cyst	16 (4.0%)	10 (2.5%)	1.62 (0.74, 3.75)	4.46 (0.92, 7.01)
Renal angiomyolipoma	9 (2.3%)	4 (1.0%)	2.28 (0.73, 8.46)	4.50 (0.94, 33.10)
Renal cyst	216 (54.0%)	167 (41.8%)	1.64 (1.24, 2.16)	1.25 (0.86, 1.81)

*Note*. The unadjusted and adjusted^*∗*^ odds ratio for patients with benign adrenocortical tumors (cases) compared with those with normal adrenal glands (controls) are presented along with 95% confidence intervals for each benign abdominal neoplasm. AOR: adjusted odds ratio; ^*∗*^adjusted for age, sex, race, smoking status, BMI, duration imaging interval, hypertension, hyperlipidemia, composite diabetes (diabetes or prediabetes), coronary artery disease, and myocardial infarction.

**Table 5:** Risk for benign neoplasia detected via other modalities.

Other benign neoplasm	Cases (adrenocortical tumor)	Controls (normal adrenal)	OR (95% CI)	AOR (95% CI)^*∗*^
*n*	400	400		
Meningioma	11 (2.8%)	5 (1.3%)	2.23 (0.80, 7.14)	2.45 (0.61, 12.42)
Pituitary adenoma	4 (1.0%)	5 (1.3%)	0.80 (0.20, 3.03)	0.64 (0.07, 4.50)
Thyroid nodule	102 (25.5%)	68 (17.0%)	1.67 (1.19, 2.36)	1.77 (1.15, 2.74)
Thyroid nodule ≥ 10 mm	66 (16.5%)	35 (8.8%)	1.88 (0.98, 3.63)	1.72 (0.73, 4.13)
FNA benign thyroid	35 (8.8%)	17 (4.3%)	1.62 (0.82, 3.29)	2.12 (0.87, 5.50)
Hyperparathyroidism or parathyroid adenoma	14 (3.5%)	5 (1.3%)	2.87 (1.08, 8.93)	3.00 (1.00, 11.64)
Benign breast Mass	16 (6.0%)	10 (3.3%)	1.62 (0.73, 3.75)	3.25 (1.28, 8.78)
Breast cyst	24 (9.0%)	46 (15.0%)	0.49 (0.29, 0.81)	0.59 (0.31, 1.10)
Colon adenoma	164 (41.0%)	136 (34.0%)	1.34 (1.01, 1.79)	1.15 (0.80, 1.65)
Benign prostatic hyperplasia	27 (20.5%)	5 (5.3%)	5.71 (2.37, 17.00)	3.20 (1.14, 10.60)
Lipoma	53 (13.3%)	46 (11.5%)	1.17 (0.77, 1.79)	1.08 (0.63, 1.83)
Fibroid	86 (32.1%)	95 (31.0%)	0.88 (0.63, 1.22)	1.36 (0.89, 2.12)
Endometrial polyp	25 (9.3%)	35 (11.4%)	0.89 (0.40, 1.79)	0.78 (040, 1.49)
Cervical polyp	22 (8.2%)	31 (10.1%)	0.69 (0.39, 1.21)	0.92 (0.46, 1.82)
Ovarian cyst	54 (20.1%)	83 (27.1%)	0.60 (0.41, 0.86)	1.40 (0.85, 2.34)

*Note*. The unadjusted and adjusted^*∗*^ odds ratio for patients with benign adrenocortical tumors (cases) compared with those with normal adrenal glands (controls) are presented along with 95% confidence intervals for each benign neoplasm. AOR: adjusted odds ratio; ^*∗*^adjusted for age, sex, race, smoking status, BMI, duration imaging interval, hypertension, hyperlipidemia, composite diabetes (diabetes or prediabetes), coronary artery disease, and myocardial infarction.

**Table 6:** Risk for malignant tumors.

Malignancy	Cases (adrenocortical tumor)	Controls (normal adrenal)	OR (95% CI)	AOR (95% CI)^*∗*^
Gastrointestinal cancer	22 (5.5%)	29 (7.3%)	0.74 (0.42, 1.32)	0.84 (0.42, 1.69)
Renal/bladder cancer	14 (3.5%)	18 (4.5%)	0.77 (0.38, 1.57)	0.98 (0.41, 2.33)
Breast cancer	36 (9.0%)	50 (12.5%)	0.69 (0.44, 1.08)	0.83 (0.47, 1.46)
Lung cancer	23 (5.8%)	12 (3.0%)	2.0 (0.97, 4.02)	1.73 (0.77, 4.10)
Brain cancer	2 (0.5%)	4 (1.0%)	0.45 (0.09, 2.73)	NA
Endocrine cancer	13 (3.3%)	11 (2.8%)	1.28 (0.53, 2.68)	1.53 (0.60, 4.11)
Hematologic cancer	15 (3.8%)	24 (6.0%)	0.61 (0.31, 1.18)	0.59 (0.27, 1.25)
Melanoma/sarcoma	4 (1.0%)	14 (3.5%)	0.28 (0.09, 0.85)	0.19 (0.05, 0.59)
Gynecologic cancer	15 (3.7%)	20 (5.0%)	0.74 (0.37, 1.47)	0.57 (0.24, 1.26)
Prostate/testicular cancer	13 (3.2%)	16 (4%)	0.80 (0.38, 1.70)	0.49 (0.16, 1.50)
Other cancer	3 (0.8%)	2 (0.5%)	1.50 (0.25, 9.04)	NA

*Note*. The unadjusted and adjusted^*∗*^ odds ratio for patients with benign adrenocortical tumors (cases) compared with those with normal adrenal glands (controls) are presented along with 95% confidence intervals for each malignancy. AOR: adjusted odds ratio; ^*∗*^adjusted for age, sex, race, smoking status, BMI, duration imaging interval, hypertension, hyperlipidemia, composite diabetes (diabetes or prediabetes), coronary artery disease, and myocardial infarction.

## Data Availability

Deidentified data supporting the conclusions of the study can be requested from the corresponding author by investigators with local institutional research ethics approval.
